# Genomic Phenotyping by Barcode Sequencing Broadly Distinguishes between Alkylating Agents, Oxidizing Agents, and Non-Genotoxic Agents, and Reveals a Role for Aromatic Amino Acids in Cellular Recovery after Quinone Exposure

**DOI:** 10.1371/journal.pone.0073736

**Published:** 2013-09-09

**Authors:** J. Peter Svensson, Laia Quirós Pesudo, Siobhan K. McRee, Yeyejide Adeleye, Paul Carmichael, Leona D. Samson

**Affiliations:** 1 Biological Engineering Department, Massachusetts Institute of Technology, Cambridge, Massachusetts, United States of America; 2 Center for Environmental Health Sciences, Massachusetts Institute of Technology, Cambridge, Massachusetts, United States of America; 3 Department of Biosciences and Nutrition, Karolinska Institutet, Huddinge, Sweden; 4 Safety & Environmental Assurance Centre, Unilever, Sharnbrook, Bedfordshire, United Kingdom; 5 Biology Department, Massachusetts Institute of Technology, Cambridge, Massachusetts, United States of America; 6 Koch Institute for Integrative Cancer Research, Massachusetts Institute of Technology, Cambridge, Massachusetts, United States of America; University of Massachusetts Medical School, United States of America

## Abstract

Toxicity screening of compounds provides a means to identify compounds harmful for human health and the environment. Here, we further develop the technique of genomic phenotyping to improve throughput while maintaining specificity. We exposed cells to eight different compounds that rely on different modes of action: four genotoxic alkylating (methyl methanesulfonate (MMS), *N*-Methyl-*N*-nitrosourea (MNU), *N,N′*-bis(2-chloroethyl)-*N*-nitroso-urea (BCNU), *N*-ethylnitrosourea (ENU)), two oxidizing (2-methylnaphthalene-1,4-dione (menadione, MEN), benzene-1,4-diol (hydroquinone, HYQ)), and two non-genotoxic (methyl carbamate (MC) and dimethyl sulfoxide (DMSO)) compounds. A library of *S. cerevisiae* 4,852 deletion strains, each identifiable by a unique genetic ‘barcode’, were grown in competition; at different time points the ratio between the strains was assessed by quantitative high throughput ‘barcode’ sequencing. The method was validated by comparison to previous genomic phenotyping studies and 90% of the strains identified as MMS-sensitive here were also identified as MMS-sensitive in a much lower throughput solid agar screen. The data provide profiles of proteins and pathways needed for recovery after both genotoxic and non-genotoxic compounds. In addition, a novel role for aromatic amino acids in the recovery after treatment with oxidizing agents was suggested. The role of aromatic acids was further validated; the quinone subgroup of oxidizing agents were extremely toxic in cells where tryptophan biosynthesis was compromised.

## Introduction

The need to develop techniques to test toxicity of chemical compounds is increasing. Ethical and legal considerations impose constraints on animal usage for compound testing, making cell culture based testing an attractive alternative. Toward this goal, we and others have developed methods for genomic phenotyping, a gene-by-gene genome-wide approach that provides mechanistic detail on the modes of action of the test compound [1]–[9] (reviewed in [10]). Routinely used tests such as the micronucleus test and mouse lymphoma assays, are prone to false positives and provide few indications of the mechanisms underlying the toxicity [11]. Further, cumbersome screens or methods prone to false positive results stress the need to develop fast, sensitive techniques for drug screening. We have previously optimized *Saccharomyces cerevisiae* genomic phenotyping for liquid assays [9] and before that, optimized the method for growth on solid agar [1], [2]. We recently showed that genomic phenotyping in yeast cells can be predictive of toxicity-modulating proteins in human cells, increasing the method’s relevance [12]. In this study, we demonstrate how high-throughput parallel sequencing can be used to enhance the power of genomic phenotyping [13]–[16].

Two of the major contributors to endogenous and exogenous DNA damage are alkylating and oxidizing agents. A review on the cellular response to DNA damage caused by alkylating agents was recently published by our group [17]. As for oxidative stress, cells are exposed to environmental oxidants, but oxidative stress also arises as a consequence of oxygen utilization for energy production and other metabolic processes [18]–[20]. The major source for reactive oxygen species (ROS) production is electrons leaked from the respiratory complexes in mitochondria. ROS are also generated by redox-active compounds such quinones and polycyclic aromatic hydrocarbons, which can be converted to redox-active quinones by aldo-keto reductases [21]. When quinones are reduced to semiquinones, superoxide (O_2_^–^) is produced and further reduction leads to hydroquinones and hydrogen peroxide (H_2_O_2_) (reviewed in [22]). By redox cycling, the three quinone products are kept in equilibrium [23]. Under normal conditions, cellular antioxidant defenses are capable of neutralizing ROS [20]. However, when ROS exceed the antioxidant capacity, they react with proteins, DNA, RNA, and lipids, which in turn may impair cellular processes [19], [20] resulting in general cell damage and apoptosis [24]. ROS have been linked to numerous diseases, particularly cancer, neurodegenerative diseases and premature aging (reviewed in [25]).

In this study we exposed a library of 4,852 strains to eight different compounds, genotoxic or non-genotoxic, alkylating or oxidizing. The library of deletion strains were grown in competition with each other and at different time points, the ratio between the strains was assessed by quantitative ‘barcode’ sequencing. The results using this method produce toxicity profiles that easily distinguish alkylating and oxidizing genotoxic compounds from each other and from non-genotoxic compounds. In addition, we show that toxicity profiles mirror previous results obtained using much more cumbersome methods, and moreover, revealed a novel role for aromatic amino acids in cellular protection after ROS-inducing agents.

## Results

### Fast and Sensitive Method for Screening Agents

**Figure 1:**
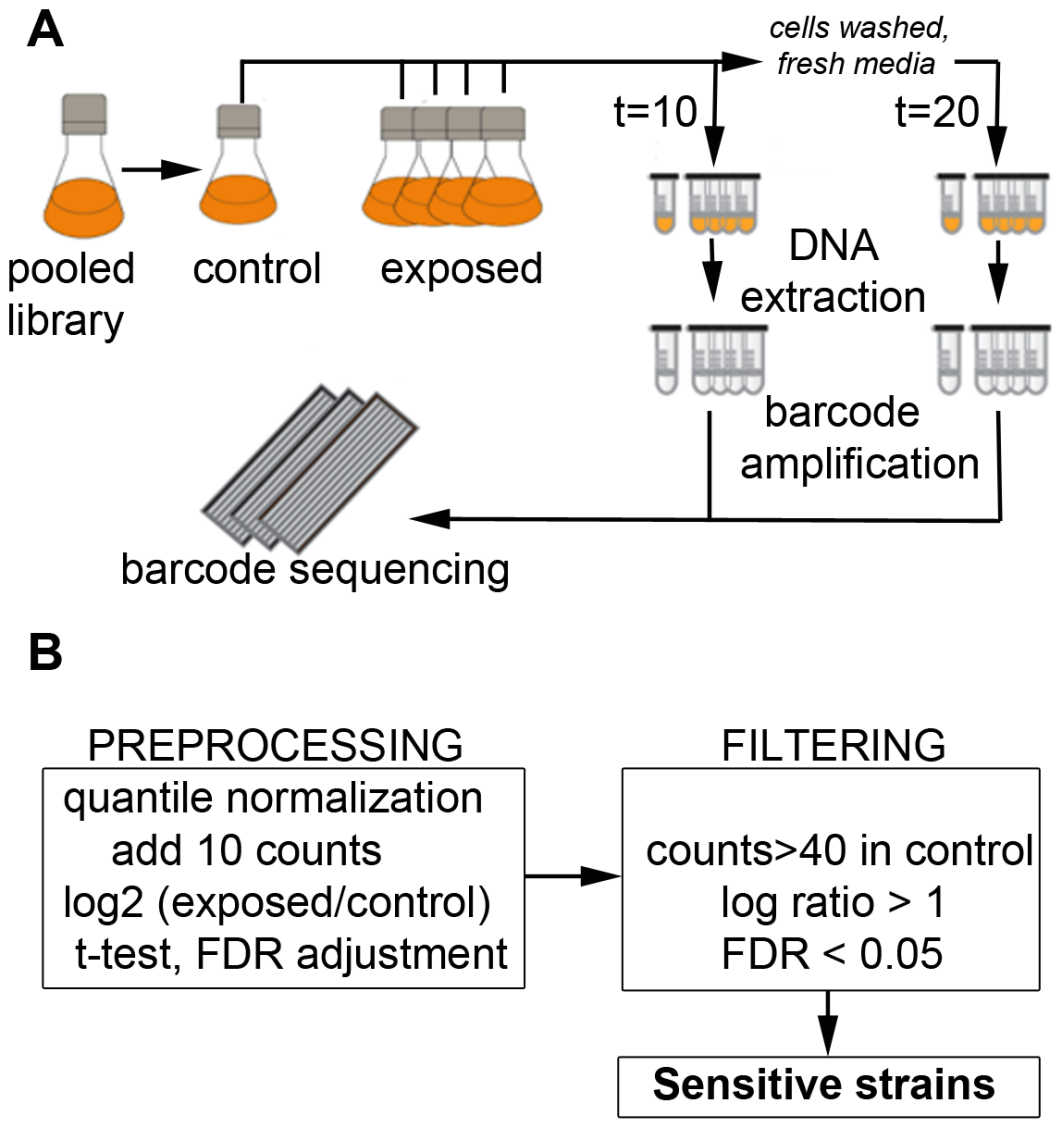
Experimental workflow for barcoded genomic phenotyping. Schematic representation of A) experimental design (t is the time of cell harvest, which was at 10 or 20 generation times) and B) analysis and filtering of high-throughput sequencing data.

To determine differential growth patterns of strains deleted for non-essential genes, cultures were made where close to equal numbers of 4,852 deletion mutant strains were pooled and then grown in competition. Each mutant has a strain-specific ‘barcode’ tag for identification. In different experiments, cultures was exposed to eight compounds: methyl methanesulfonate (MMS), *N*-methyl-*N*-nitrosourea (MNU), *N,N′*-bis(2-chloroethyl)-*N*-nitrosourea (BCNU), *N*-ethylnitrosourea (ENU), 2-methylnaphthalene-1,4-dione (menadione, MEN), benzene-1,4-diol (hydroquinone, HYQ), methyl carbamate (MC) and dimethyl sulfoxide (DMSO). Seven of these compounds are carcinogens: the alkylating agents MMS, MNU, BCNU and ENU, plus the oxidizing agents MEN and HYQ; whereas MC is a non-genotoxic carcinogen. The eighth compound, DMSO, was a control, which is neither genotoxic nor carcinogenic (Table 1). Cultures were exposed to 4–5 doses for each agent. Exposure was chronic for 10 generation times, as measured by increased OD_595_ for each individual culture, at which point a sample was taken for sequencing. For the remaining culture, growth media was exchanged with fresh media without the test compound and the cells were grown for another 10 generation times to recover, after which a second sample was taken for sequencing. After DNA extraction from each sample, the representation of each strain was determined by Illumina high-throughput sequencing of the strain-specific barcoded tags (Figure 1A).

**Table 1:** List of selected compounds and doses used in this study.

Compound	Abbreviation	CAS number	Group	Dose (mM)
Methyl methanesulfonate	MMS	66-27-3	Alkylating agent, genotoxic carcinogen	18.13, 36.32, 54.48, 72.64
N-Methyl-N-nitrosourea	MNU	684-93-5	Alkylating agent, genotoxic carcinogen	0.48, 0.97, 1.46, 1.94
*N*-Ethyl-*N*-nitrosourea	ENU	759-73-9	Alkylating agent, genotoxic carcinogen	1.708, 3.416, 5.124, 6.832
*N,N′*-bis(2-chloroethyl)-*N*-nitroso-urea	BCNU	154-93-8	Alkylating agent, genotoxic, therapeuticchemical	0.028, 0.056, 0.084, 0.112
2-Methylnaphthalene-1,4-dione(Menadione)	MEN	58-27-5	Oxidizing agent, genotoxic, therapeuticchemical	0.005, 0.010, 0.015, 0.020
Benzene-1,4-diol (Hydroquinone)	HYQ	123-31-9	Oxidizing agent, genotoxic, therapeutic chemical	18.164, 22.705, 27.245, 31.786
Methyl carbamate	MC	598-55-0	Non-genotoxic carcinogen	0.028, 0.056, 0.084, 0.112
Dimethyl sulfoxide	DMSO	67-68-5	Non-genotoxic, no carcinogen	0.5, 0.6, 0.7, 0.8, 0.9

To determine the individual strain fitness, the sequencing data was first filtered in several steps (Figure 1B): (i) by absolute number of barcode sequence counts (>40); (ii) by fold change compared to untreated control (abs(log_2_) >1) for each dose; and (iii) by statistical significance (Student’s t-test followed by Benjamini-Hochberg False Discovery Rate (BH-FDR), BH-FDR <0.05). Each compound generated up to 230 sensitive strains (Table S1). 3,807 out of 4,994 strains (76%) passed the quality criteria of being represented by at least 40 sequencing reads in at least one of the untreated samples. 1,203 strains (32%) showed reduced fitness when grown in at least one of the compounds (Table S2) and a single strain (*pol32*Δ) was sensitive to all eight agents. The high number of sensitive strains is expected given that almost all genes are required under certain conditions [6]. There were few differences between the growth for 10 or 20 generation times.

### Genotoxic Agents have Different Profiles of Proteins Needed for Survival

**Figure 2:**
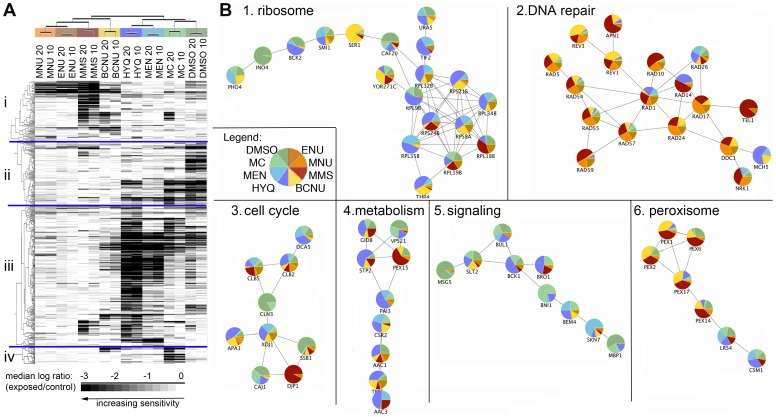
The difference between alkylating and oxidizing agents can be explained by fitness profiles of the strains. A) Two-dimensional hierarchical clustering of fitness ratio (median log ratio of exposed/control) results using the strains sensitive after 10 and 20 generation times upon exposure to different chemicals. Compounds and doses are plotted across the horizontal axis. On the vertical axis, a subset of 508 strains with reduced fitness is shown. B) Protein-protein interaction networks with >5 toxicity-modulating proteins. The colors (explained in legend, same as labels in A) within the pie charts indicate the contribution of each of the eight compounds. Alkylating agents represented in shades of yellow-red, oxidizing agents in shades of blue and non-genotoxic compounds in green.

To group the agents by the gene products needed for cell growth in the presence of that agent, we performed hierarchical clustering of the fitness profiles. Strains that showed reduced fitness to at least one of the compounds were included in the clustering (Figure S1). The fitness so far was recorded as the log_2_ ratio between exposed and unexposed cells. Here, the fitness was summarized into a score representing the median of the log ratios of the four-five doses for each compound. In parallel, we used two groups, the entire set of 1,203 sensitive strains and a subset of 508 sensitive strains (Table S3). The smaller set used more stringent selection criteria by limiting the input from each compound to a maximum of 20 toxicity-modulating strains per dose, time-point and replicate. This threshold was selected as the median number of toxicity-modulating strains per data point was 23 (Figure S2). By this method we achieve an easier overview of the data as well as leveling the contributions of the different compounds and avoid a clustering based on a single compound which might arise as a technical artifact. Unsupervised hierarchical clustering of the fitness scores revealed that, on the horizontal compound axis, the main break in the clustering tree was created by alkylating (MNU, ENU, MMS, BCNU) versus non-alkylating agents (Figure S1, Figure 2A). Non-alkylating agents were further divided into oxidizing (HYQ, MEN) and non-genotoxic (MC, DMSO) agents. On the vertical strain axis of the stringent cluster (Figure 2A), four main clusters were formed by the data-driven clustering (i-iv). Cluster (i) deviated the most from the rest and was associated specifically with reduced fitness after exposure to the four alkylating agents. The largest cluster (iii) was comprised of strains sensitive primarily to the oxidizing agents, whereas two clusters (ii, iv) were associated with strains showing growth retardation after exposure to non-genotoxic agents (Table S3). The 31 strains in cluster (iv) have deletions of proteins implicated in a wide spectrum of biological functions, including microtubule processing (Smy1, She1, Jnm1, Num1, Bni1) and protein deubiquitination (Ubp2, Ubp8, Sgf11).

To further characterize the strains, the protein-protein interactions between the proteins that were absent in each of the sensitive strains were mapped (Figure S3). The dense network of all proteins leading to sensitivity when deleted was reduced by considering the more stringent selection (Figure 2B). From the protein-protein interaction networks of this set of strains with reduced fitness, we identified seven interconnected components with more than five nodes. The largest component with 19 nodes (Figure 2B1) consisted of subunits of the ribosomal complex. The major contribution came from proteins required for recovery upon exposure to oxidizing agents, but some of these proteins also contributed toward the recovery upon exposure to alkylating agents. The second largest component with 17 nodes (Figure 2B2) consisted almost exclusively of DNA repair proteins. In this network, proteins required for recovery upon exposure to the alkylating agents made up the entire cluster. For the four remaining networks (Figure 2B3–7), contributions were for recovery from both genotoxic and non-genotoxic compounds and the functions represented were (broadly defined) cell cycle regulation, metabolism, signaling and peroxisome organization.

### Classification of Toxicity-modulating Proteins into Functional Groups

**Figure 3:**
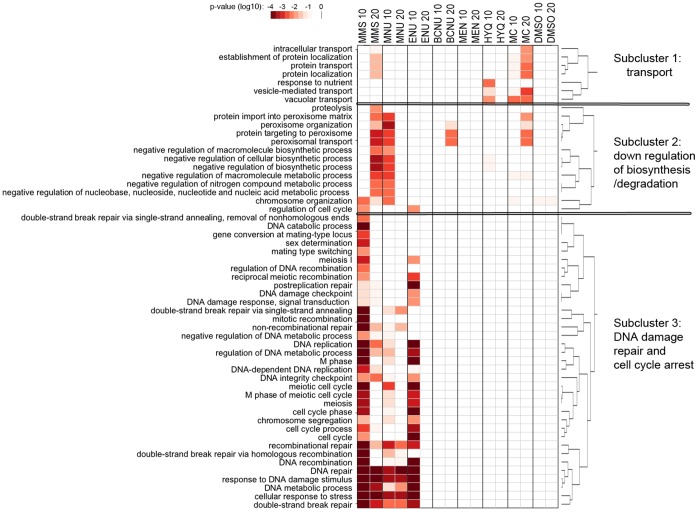
Functional enrichment reveals an alkylating agent-specific DNA repair and cell cycle dependency. Gene-annotation enrichment analysis heat map and clustering for sensitive strains to different compounds at early (10 generation times) or late (20 generation times) timepoints. Heat map colors correspond to the –log10 of the p-values.

To look for enrichment of functional categories among the proteins that contribute to damage recovery, we analyzed the lists with respect to gene ontology terms or KEGG pathways (Figure 3, Table S4). The functional categories affected by the eight compounds (FDR<0.05) can be divided into three subclusters: (1) transport, (2) degradation/down regulation of biosynthesis, and (3) DNA damage repair and cell cycle arrest (Figure 3). This third cluster contains the most enriched subcluster composed of DNA repair proteins needed for recovery upon exposure to the alkylating agents MMS, MNU and ENU, and also a second subcluster of cell cycle and DNA replication are required for recovery from exposure to the same agents. Recovery from exposure to oxidizing agents requires vesicle transport and autophagy proteins to a higher degree compared to recovery from alkylation damage.

In the initial phase, during the first 10 generation times (Table S4), DNA repair and cell cycle-related GO or KEGG terms are prevalent after treatment with genotoxic agents. For the non-alkylating agents, the enriched GO terms involve vacuolar transport and, surprisingly, for the oxidizing agents, phenylalanine, tyrosine and tryptophan biosynthesis are also represented. The involvement of aromatic amino acid synthesis has not, to our knowledge, previously been linked to the response to oxidative stress.

In the recovery phase scored after 20 generation times (Table S4), the MMS and MNU exposed strains lacking different aspects of DNA repair continue to remain depleted whereas the strains compromised for cell cycle control show increased representation. Interestingly, the strains that only appear as having reduced fitness in the recovery phase are largely involved in different aspects of transport and relocalization of different cellular components. Under the less stringent conditions (non-adjusted p<0.1) we re-identify the biosynthesis of aromatic amino acids as influencing recovery after exposure to the oxidizing agents MEN and HYQ. The strains that showed altered growth in DMSO produced no enriched GO terms at the higher significance level (FDR<0.05). At lower stringency level (non-adjusted p<0.1) of the enrichment (Tables S5 and S6), an expanded view can be seen.

### Aromatic Amino Acids Specifically Required After ROS Exposure

**Figure 4:**
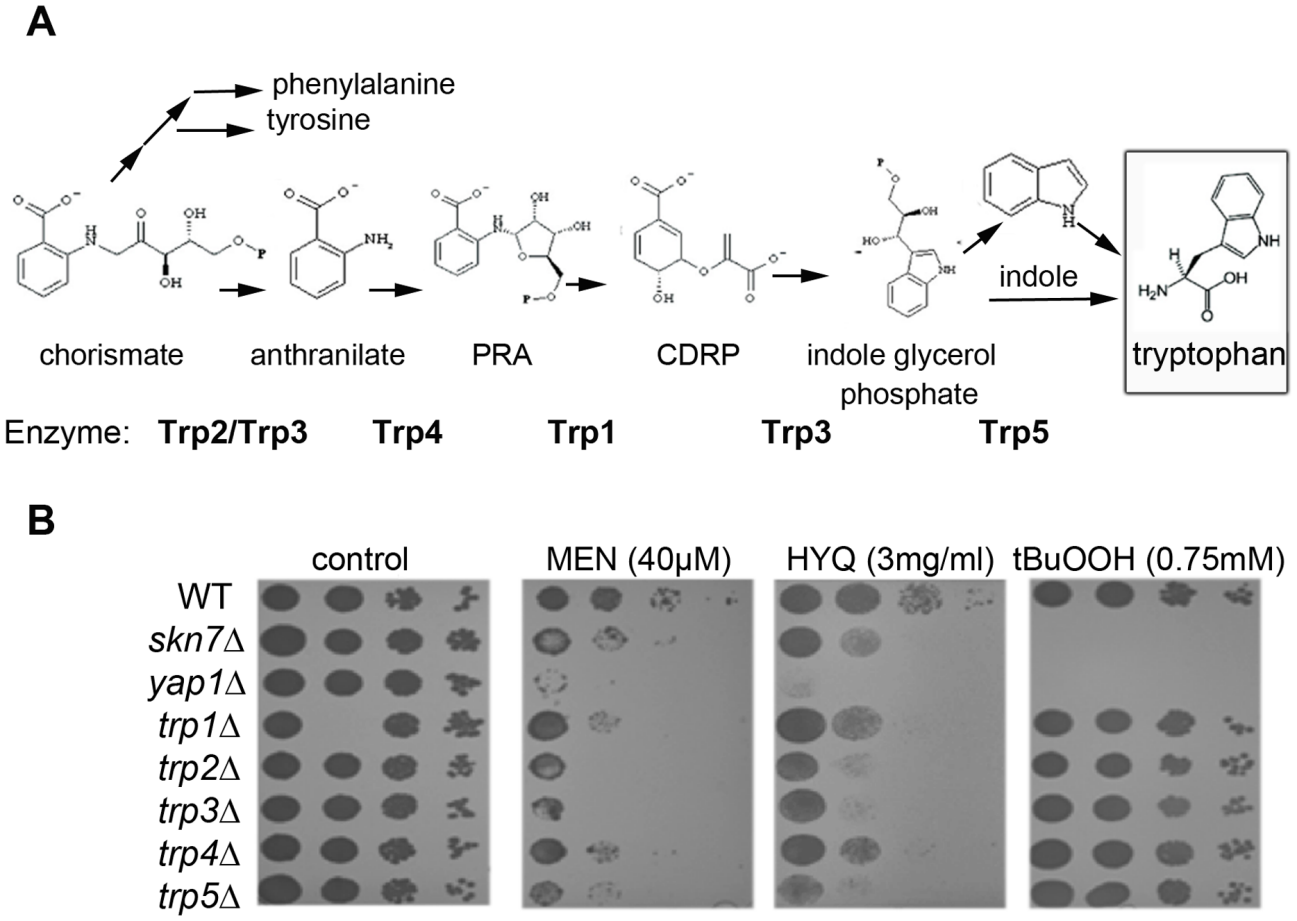
Tryptophan biosynthesis rescues cells from ROS. A) A schematic of the tryptophan biosynthesis pathway [29]. PRA: N-(5′-phospohribosyl)-anthsranilate, CDRP: 1-(o-carboxyphenylamino)-1-desoxyribuose-5-phosphate. B) Compound sensitivity of selected mutant strains were analyzed by spot assay. Strains were grown in liquid YPD+G418 overnight at 30°C and then diluted in YPD. Ten-fold serial dilutions of each yeast culture was spotted onto YPD plates in the absence (control) and presence of the different compounds: MMS (0.006%), MEN (40 µM), HYQ (3 mg ml^−1^), and tBuOOH (0.75 mM). Plates were incubated at 30°C and growth was recorded after 48 h exposure.

To validate the requirement for the biosynthesis of aromatic amino acids, and especially tryptophan synthesis, for cells to recover after oxidative genotoxic stress, we exposed five different strains lacking various components of the tryptophan biosynthesis pathway to both ROS-generating (HYQ and MEN) and non-ROS-generating (tBuOOH) oxidative stress (Figure 4). The requirement for tryptophan synthesis turns out to be specific for recovery upon exposure to the ROS-generating quinones/hydroquinones (MEN, HYQ). Tryptophan synthesis does not influence recovery from the differently acting oxidizing agent tBuOOH. As controls we used cells lacking transcription factors Skn7 and Yap1 that are needed for the transcription of oxidative stress responsive genes [26]. As expected, the *skn7* and *yap1* strains were sensitive to both quinone and non-quinone oxidizing agents.

### Comparison with Previous Studies

To estimate the specificity and sensitivity of the barcode-sequencing method described here we compared the MMS results to those obtained in previous extensive genomic phenotyping studies, both from our group on solid agar [2] and in liquid media [9] and others [7], [27]. Of the 48 strains that were associated with reduced fitness upon exposure to MMS for 10 generation times in this study, 43 (90%, p = 7×10^−18^ Fisher’s exact test) were also identified as MMS sensitive when grown on solid agar. Among the strains that showed a growth defect during the recovery phase (20 generation times), 147 of 226 (65%, p = 2×10^−29^) were identified in the solid agar assay. In comparison with the genomic phenotyping in liquid media, 24 of the strains (50%, p = 1.4×10^−12^) identified at 10 generation times and 35 of the strains identified at 20 generation times (15%, p = 0.006) in this study were recovered in the previous results. These results indicate that the specificity is high using the barcode-sequencing method.

As for the sensitivity, this method identified in total 240 strains as MMS-sensitive, whereas in the solid agar assay, 1,455 strains were identified as MMS sensitive [2] and in the liquid media assay, 479 strains were identified as MMS sensitive [9], giving a recovery rate of 16% compared to the solid agar assay and 50% compared to liquid media assay. However, when stratifying the data into categories of different sensitivity, we note that of the most sensitive strains in the solid agar screen (with a sensitivity score 30; see ref [2]), 43% (13/30) are also identified using barcode-sequencing. For the highly sensitive strains (score >20), 34% (26/76) are recovered and for the strains with medium sensitivity (score between 11 and 20), 25% (48/195) are recovered. Only for the strains showing low sensitivity in the solid agar screen (score ≤10) is the recovery rate poor, as only 7.4% (88/1184) of the strains are identified using the barcode-sequencing approach.

Comparison with data from other groups also shows consistency between results. Among the proteins causing MMS-sensitivity when deleted, 73% (35/48) are also found in study from the Parsons *et al* [7], and from an earlier study from Chang *et al* [27], 38% (18/48) are re-found in the list of proteins needed for MMS resistance. The lower concordance with the latter study, is consistent with a similarly low recovery between these two studies, 38% (39 out of 102) MMS-sensitive strains from Parsons et al [7] are also identified in Chang *et al* [27].

Together, the data from these comparisons indicate that the barcode-sequencing method identifies toxicity-modulating proteins with high confidence.

## Discussion

Here we present a screening method for drug toxicity with data for eight compounds. The technique separates out different subsets of proteins needed for cellular recovery upon exposure to a range of genotoxic and non-genotoxic compounds. This study targets a problem that faces pharmaceutical and other industries requiring early indicators of the genotoxic effects of a test compound. The solid agar genomic phenotyping previously developed [1], [2], provided a sensitive and robust method to screen a library of eukaryotic cells with a test compound in order to ascertain whether the compound is toxic and if so by what mechanism. However, the method uses individually grown cultures of each mutant strain and is too cumbersome to be a technically feasible approach for systematic screening of large numbers of compounds. The method presented here provides a realistic alternative, with maintained specificity, although with a lower sensitivity.

The results presented in this study provide comprehensive profiles of the proteins required for cellular recovery after exposure to a range of alkylating, oxidizing and also non-genotoxic compounds. Many of the identified toxicity-modulating proteins and pathways, such as the DNA repair cluster needed after alkylating agent treatment, have already been heavily studied [28]. However, novel pathways needed for recovery also came to light, such as the requirement of aromatic amino acid synthesis (reviewed in [29]) following exposure to the oxidizing agents menadione (MEN) and hydroquinone (HYQ). Amino acid uptake or synthesis has previously been implicated in survival after a variety of compounds [30]–[33]. A question that remains unanswered is, what is the role of aromatic amino acid synthesis in cellular recovery specifically after quinone exposure? It has been reported that charge transfer can occur between semiquinones and the aromatic amino acids tyrosine and tryptophan [34]. Possibly, the funneling of unpaired electrons to the free aromatic acids acts as a scavenger for oxygen radicals produced by the quinone/hydroquinone redox cycling [35], [36]. It was previously known that the transcriptional profile of H_2_O_2_ and MEN exposed cells are largely similar [37]. Genes, such as superoxide dismutases, glutathione peroxidases, and thiol-specific antioxidants, involved in the detoxification of both H_2_O_2_ and O_2_^−^ are strongly induced after exposure. The majority of these genes are under control of the Yap1 and Skn7 transcription factors [38]. However, the Skn7 and Yap1 proteins are also activated in the response to tBuOOH, suggesting that the tryptophan synthesis is induced under a different system.

As the details of the DNA damage response are being elucidated, it is becoming evident that many pathways other than DNA repair and cell cycle checkpoints are involved in cellular recovery after exposure to DNA damaging agents [1], [2], [9], [12]. At the time of damage, several different sets of proteins are needed for a plethora of functions to deal with several different kinds of molecular damage, but once the cell has dealt with that damage, the cell reinitiates the cell cycle, requiring another set of proteins. Some genes, such as the environmental stress response (ESR) genes [37], are transcriptionally regulated independently of the stressor. Expression of many other genes is stressor specific. Among the 868 ESR proteins, 23 (8%) were identified in this study, indicating that the toxicity-modulating proteins identified by genomic phenotyping complements the results from expression profiling.

In conclusion, while further developing a higher throughput method for toxicity screening, we have discovered that biosynthesis of aromatic amino acids, and specifically tryptophan synthesis, provides protection from quinone/hydroquinone-induced ROS in eukaryotic cells.

## Materials and Methods

### Reagents

Test componds were purchased from Sigma-Aldrich: MMS (Cat #129925), MNU (Cat #N4766), menadione (Cat #M5625), hydroquinone (Cat #240125), methyl carbamate (Cat #246352), DMSO (Cat #D2650), BCNU (Cat #C0400), ENU (Cat #N8509).

### Deletion Pool Construction, Cell Culture, and Sample Preparation

Pools of the yeast haploid deletion collection were prepared as described previously [13]. The deletion pools were cultured at 30°C and 250 rpm in an incubator shaker using YPD media (10 g/l yeast extract, 20 g/l peptone, 20 g/l dextrose) containing 100 µg/ml G418. To prepare sequencing samples, we followed the protocol as described previously [14], [39]. DNA was isolated using YeaStar genomic DNA kit (ZymoResearch Inc, Irvine CA). Barcodes were PCR-amplified and the resulting PCR-fragments were sequenced at the BioMicroCenter at MIT.

### Sequence Data Processing

Analysis was performed similarly to as previously described [40]. Samples were sequenced on a Solexa Genome Analyzer 2.0, generating single ends reads of 35 nucleotides. The first four bases comprise the multiplexing code used to assign each read to an experiment and a sample (five total for each experiment: one of four sorted fractions or the original pool). The next set of bases identify whether the read corresponds to an uptag or downtag barcode, and the final 14 bases (13 for uptags) provides sufficient information to uniquely identify the barcode [14]. Custom Matlab scripts parse sequencing reads to tabulate the number of perfect barcode reads for each strain in each sample, and for each experiment.

The tabulated counts for each strain were normalized to a frequency for each sample as to compare frequencies in the exposed cultures to the frequencies in the original (unexposed) pool. These frequencies are calculated separately for uptags and downtags, as well as for each independent experiment (i.e. each screen has two independent replicates, so that there are four separate frequency vectors for each sample in each screen). Because each sample contains strains for which few or no barcodes are detected, a baseline value of 10 counts was added to each vector of frequencies to eliminate spurious hits arising from poorly represented strains. The raw counts were quantile-normalized and then treated/control ratio was log-transformed (base 2).

### Data Analysis

Custom scripts were written in R. Cluster and Treeview [41], Cytoscape [42], GoogleCharts was used for visualization. Raw data is deposited at SRA with accession number SRA091991.

## Supporting Information

Figure S1 **Two-dimensional hierarchical clustering of fitness ratio (median log ratio of exposed/control) results using the strains sensitive after 10 and 20 generation times upon exposure to different chemicals.** Compounds and doses are plotted across the horizontal axis. On the vertical axis, the 1,203 strains with reduced fitness are shown.
(TIF)

Figure S2 **Histogram of number of sensitive strains for each data point (in total 160).** Red hashed line indicates the median number (23) of strains at each data point.
(TIF)

Figure S3 **Protein-protein interaction network of all 1,203 toxicity-modulating strains.**
(TIF)

Table S1 **Growth retarded strains.**
(XLSX)

Table S2 **Log ratio (treated/control) of growth retarded strains (all).**
(XLSX)

Table S3 **Log ratio (treated/control) of growth retarded strains (top 20).**
(XLSX)

Table S4 **Enriched gene sets after 10 and 20 generation times (stringent criteria, FDR<0.05).**
(XLSX)

Table S5 **Enriched gene sets after 10 generation times (complete lists).**
(XLSX)

Table S6 **Enriched gene sets after 20 generation times (complete lists).**
(XLSX)
