# Supplementary figures and images for: Genomic Phenotyping by Barcode Sequencing Broadly Distinguishes between Alkylating Agents, Oxidizing Agents, and Non-Genotoxic Agents, and Reveals a Role for Aromatic Amino Acids in Cellular Recovery after Quinone Exposure
Source: PLoS One. 2013 Sep 9;8(9):e73736. doi: 10.1371/journal.pone.0073736 (PMC3767620; doi:10.1371/journal.pone.0073736)

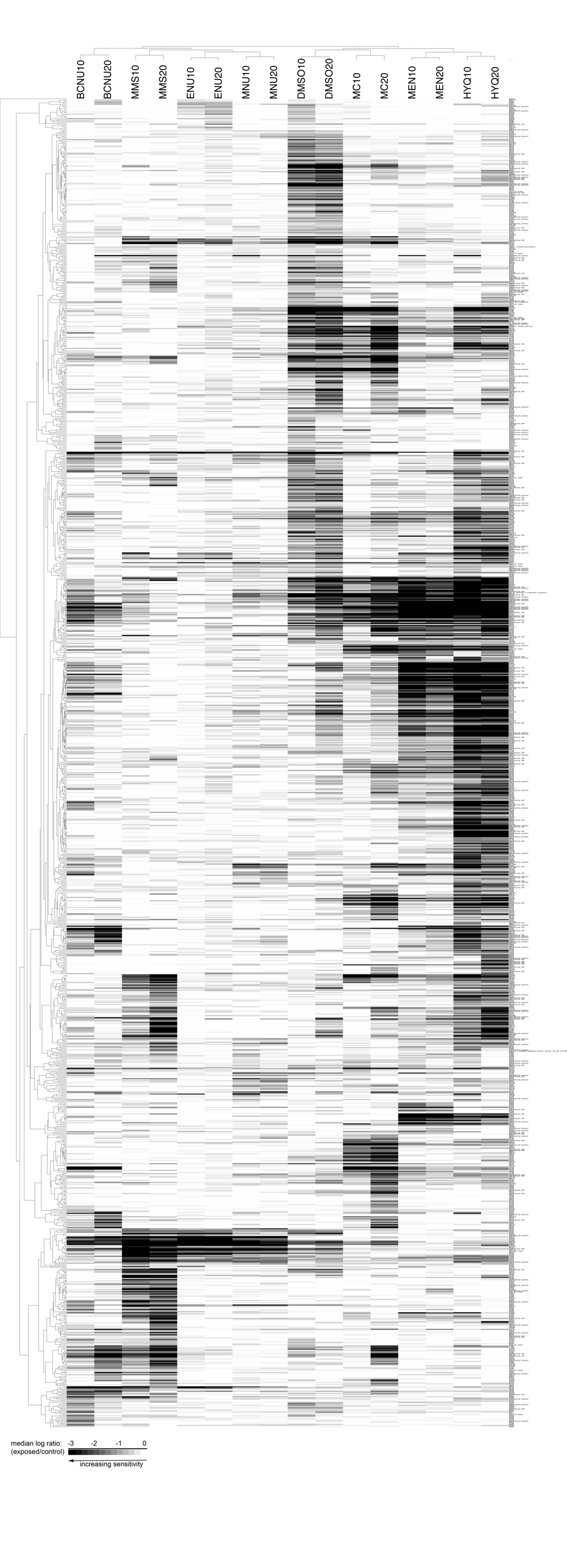

Supplement: Figure S1 — Two-dimensional hierarchical clustering of fitness ratio (median log ratio of exposed/control) results using the strains sensitive after 10 and 20 generation times upon exposure to different chemicals. Compounds and doses are plotted across the horizontal axis. On the vertical axis, the 1,203 strains with reduced fitness are shown. (TIF) [file pone.0073736.s001.tif]

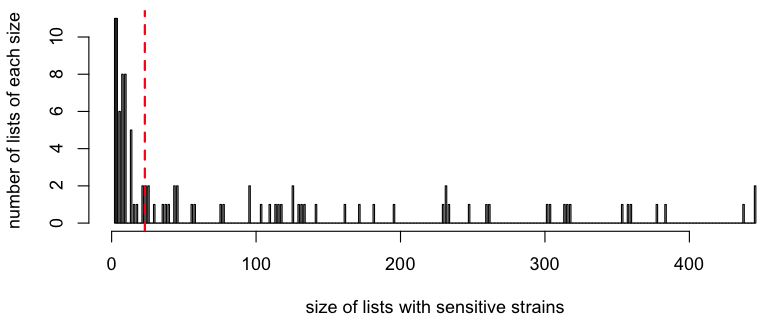

Supplement: Figure S2 — Histogram of number of sensitive strains for each data point (in total 160). Red hashed line indicates the median number (23) of strains at each data point. (TIF) [file pone.0073736.s002.tif]
